# Texture Analysis of ^18^F-FDG PET/CT for Differential Diagnosis Spinal Metastases

**DOI:** 10.3389/fmed.2020.605746

**Published:** 2021-01-15

**Authors:** Xin Fan, Han Zhang, Yuzhen Yin, Jiajia Zhang, Mengdie Yang, Shanshan Qin, Xiaoying Zhang, Fei Yu

**Affiliations:** ^1^Department of Nuclear Medicine, Shanghai Tenth People's Hospital, Tongji University School of Medicine, Shanghai, China; ^2^Shanghai Clinical College, Anhui Medical University, Shanghai, China

**Keywords:** spinal metastases, texture analysis, PET/CT, diagnosis, machine learning

## Abstract

**Purpose:** To evaluate the value of texture analysis for the differential diagnosis of spinal metastases and to improve the diagnostic performance of 2-deoxy-2-[fluorine-18]fluoro-D-glucose positron emission tomography/computed tomography (^18^F-FDG PET/CT) for spinal metastases.

**Methods:** This retrospective analysis of patients who underwent PET/CT between December 2015 and January 2020 at Shanghai Tenth People's Hospital due to high FDG uptake lesions in the spine included 45 cases of spinal metastases and 44 cases of benign high FDG uptake lesions in the spine. The patients were randomly divided into a training group of 65 and a test group of 24. Seventy-two PET texture features were extracted from each lesion, and the Mann-Whitney *U*-test was used to screen the training set for texture parameters that differed between the two groups in the presence or absence of spinal metastases. Then, the diagnostic performance of the texture parameters was screened out by receiver operating characteristic (ROC) curve analysis. Texture parameters with higher area under the curve (AUC) values than maximum standardized uptake values (SUVmax) were selected to construct classification models using logistic regression, support vector machines, and decision trees. The probability output of the model with high classification accuracy in the training set was used to compare the diagnostic performance of the classification model and SUVmax using the ROC curve. For all patients with spinal metastases, survival analysis was performed using the Kaplan-Meier method and Cox regression.

**Results:** There were 51 texture parameters that differed meaningfully between benign and malignant lesions, of which four had higher AUC than SUVmax. The texture parameters were input to build a classification model using logistic regression, support vector machine, and decision tree. The accuracy of classification was 87.5, 83.34, and 75%, respectively. The accuracy of the manual diagnosis was 84.27%. Single-factor survival analysis using the Kaplan-Meier method showed that intensity was correlated with patient survival.

**Conclusion:** Partial texture features showed higher diagnostic value for spinal metastases than SUVmax. The machine learning part of the model combined with the texture parameters was more accurate than manual diagnosis. Therefore, texture analysis may be useful to assist in the diagnosis of spinal metastases.

## Introduction

The spine is the third most common site of metastatic disease after the lungs and liver, with ~60–70% of patients with systemic cancer developing spinal metastases (1). Early and correct diagnosis of spinal metastases is helpful to guide clinical treatment, improve prognosis, and increase the survival rate. Computed tomography (CT) and magnetic resonance imaging (MRI) have their own advantages and disadvantages with regards to the diagnosis of spinal metastases. Two-deoxy-2-[fluorine-18]fluoro-D-glucose (^18^F-FDG) imaging is more sensitive than conventional imaging examinations. It can facilitate early detection of the lesion by monitoring changes in glucose metabolism and can determine metastases of both the whole body skeletal system and soft tissues in a single examination. However, there is a certain degree of false-positive influence on the diagnosis, such as trauma-induced vertebral fractures, bone hyperplasia, and metabolic bone disease, which may interfere with the diagnosis.

The maximum standardized uptake value (SUVmax) is an intuitive quantitative measure of tissue ^18^F-FDG uptake in current positron emission tomography/computed tomography (PET/CT) diagnostics. However, the currently established SUVmax diagnostic threshold has no clear criteria for spinal metastases. SUVmax is easy to use but does not fully reflect tumor size or tumor heterogeneity (2). Therefore, it is important to use texture analysis to extract more parameters in order to improve the diagnostic accuracy of metastatic lesions.

Texture analysis is a set of computational methods that extracts information regarding the relationship between adjacent pixels or voxels and assesses inhomogeneity, which can reflect the degree of benign or malignant properties and pathological features of the tissue (3, 4). Current applications are mainly based on CT and MRI, while lesser applications are applied to texture analysis using PET. Texture analysis using PET is more closely related to biological activity than texture parameters derived from CT and MRI. Furthermore, recent studies have shown that the grayscale texture variation characteristics of tumors in PET images can be used to evaluate the amount and unevenness of FDG uptake, which can be used to quantify the degree of tumor heterogeneity. For example, several studies have demonstrated that texture analysis can be used to find more powerful imaging biomarkers highly relevant to modern cancer therapy. Moreover, personalized treatment can be achieved through non-invasive molecular and genomic mapping of tumors (5–7).

In this study, spinal metastases or benign high ^18^F-FDG uptake lesions were selected to analyze the diagnostic value of texture parameters derived from adjunctive PET/CT for spinal metastases. The purpose of this study was to initially verify whether texture analysis has a diagnostic value for spinal metastases and to screen out texture parameters that have better clinical guidance for the diagnosis of spinal metastases than those of traditional diagnostic methods.

## Materials and Methods

### Study Population

This retrospective analysis was approved by the Ethics Committee of the Shanghai Tenth People's Hospital (SHSY-IEC-4.1/20-150/01) and registered with the Chinese Clinical Trials Registry (ChiCTR2000038089). We collected ^18^F-FDG PET/CT image data of patients who had a positive spinal uptake of FDG and were admitted to the Shanghai Tenth People's Hospital between December 2015 and January 2020. Inclusion criteria were as following: complete electronic medical records or clear histopathological confirmation of spinal metastases, PET/CT image quality meeting diagnostic requirements, and basic clinical information available. Exclusion criteria were as following: primary spinal tumor, treated patients, tumor margins too difficult to delineate, incomplete clinical information, image quality not meeting diagnostic requirements, irregular spine, and inability to complete the follow-up. Patients with confirmed metastases all had not received antineoplastic therapy and either had undergone at least 6 months of follow-up or ended in death, moreover, patients with benign spinal uptake of FDG had no history of tumor.

### Scanner and Acquisition Protocol

Image acquisition was performed using uMI 510 PET/CT. ^18^F-FDG was manufactured by the Shanghai Xinke Pharmaceutical Company. The patient was fasted for more than 6 h before the examination, and the blood glucose was controlled <11.0 mmol/L, patients were administered ^18^F-FDG at a dose of 0.10–0.15 mCi/Kg as the standard, and rested calmly for 60 min after intravenous injection of ^18^F-FDG. The patient was placed in supine position during the scan, and the scanning range was from the skull base to the upper 1/3 of the femur. The PET images were reconstructed using the ordered subset maximum expected value iterative method (OS-EM), with image attenuation correction using CT scan data. The images were transferred to an Ulead workstation for frame-to-frame image alignment and fusion display.

### Image Analysis

#### Data Grouping

The gold standard for the diagnosis of spinal metastases was clinical follow-up or pathologic confirmation of the lesion. The diagnosis was made by two nuclear medicine physicians with more than 10 years experiences in PET/CT diagnosis, without providing the patient's medical history or clinical data. The main criteria for manual diagnosis were osteolytic, osteogenic, or mixed bone lesions on PET/CT fusion images, partially accompanied by soft tissue mass formation, and abnormal increasing of FDG metabolism in the corresponding areas.

#### Extraction and Analysis of Texture Features

A nuclear medicine physician with more than 5 years of experience in PET/CT diagnosis sketched the high FDG metabolic lesions. A total of 6,408 heterogeneity indices were extracted from 72 PET texture features to evaluate the diagnostic value of PET texture analysis for spinal metastases. Six belonged to the co-occurrence matrix (C), 11 to the Voxel-alignment matrix, five to the neighborhood intensity-difference (NID), 11 to the intensity-size-difference zone matrix (ISZ), seven to the normalized co-occurrence (NC), 13 to the voxel statistics, two to the texture spectrum, three to the texture feature coding, nine to the texture feature coding co-occurrence, five to the neighborhood gray level dependence (NGLD) matrix. Detailed characterizations have been reported in previous studies (8).

#### Diagnostic Model Construction and Evaluation

We randomly divided the patients into a training group and a test group using the R 4.0.2 and set the seed number to 300. The construction of the machine learning model was based on the Python 6.3 platform. Moreover, the three machine learning models, logistic regression, support vector machine, and decision tree were trained and tested using the sklearn package. In both training group and testing group, the logistic regression model was compared with the diagnostic performance of SUVmax by plotting receiver operating characteristics (ROC) on the probability output of the training group.

### Statistical Analysis

Analysis was performed using the SPSS 23.0 (IBM Statistics, New York, USA) and MedCalc Statistical Software version 15.2.2 (MedCalc Software bvba, Ostend, Belgium; http://www.medcalc.org; 2015) for analysis. Data conforming to a normal distribution are described as mean ± standard deviation (SD) of the overall distribution, otherwise as interquartile range (IQR). Data from the texture analysis were subjected to the Mann-Whitney *U*-test, and parameters with statistically significant differences were selected to plot the ROC curve and calculate the area under the curve (AUC). Spearman's test was used to analyze the correlation between texture parameters. DeLong's test was used to determine the difference between the ROC curves. The Kaplan-Meier method and Cox regression were used for single-factor and multi-factor survival analysis, respectively, and *P* < 0.05 was considered to be statistically significant.

### Survival Analysis

The follow-up cutoff date was August 10, 2020; the endpoint event was patient death, and the follow-up period ranged from 3 to 40 months with an average follow-up time of 10.7 months. Single-factor and multi-factor survival analysis and mapping were performed using the R 4.0.2.

## Result

### Basic Patient Information

A total of 45 patients with confirmed spinal metastases and 44 patients with positive spinal uptake of FDG were analyzed, outlining a total of 89 lesions. After randomization into either the training or test group, the basic information and statistical differences between the two groups were compared, as shown in Table 1.

**Table 1:** Clinical characteristics of patients in training and test groups.

	**Training cohort (*****n*** **= 65)**			**Test cohort (*****n*** **= 24)**				
	**Tumor (*n* = 33)**	**Normal (*n* = 32)**	***P***	**Tumor (*n* = 12)**	**Normal (*n* = 12)**	***P***	***P_***a***_***	***P_***b***_***
Age (years)			0.106			0.181	0.100	0.399
Mean ± SD	64.67 ± 8.324	59.44 ± 16.274		60.25 ± 5.956	63.67 ± 8.669			
Range	39–80	27–84		52–73	52–81			
Sex			0.015			0.041	0.787	0.323
Male	26	16		9	4			
Female	7	16		3	8			

*The P_a_ was derived from the Student's t or chi-square test of tumor groups between the training and test cohorts and the P_b_ was derived from that of normal groups between the training and test cohorts*.

### Distinction Between Tumor and Normal Group

**Figure 1:**
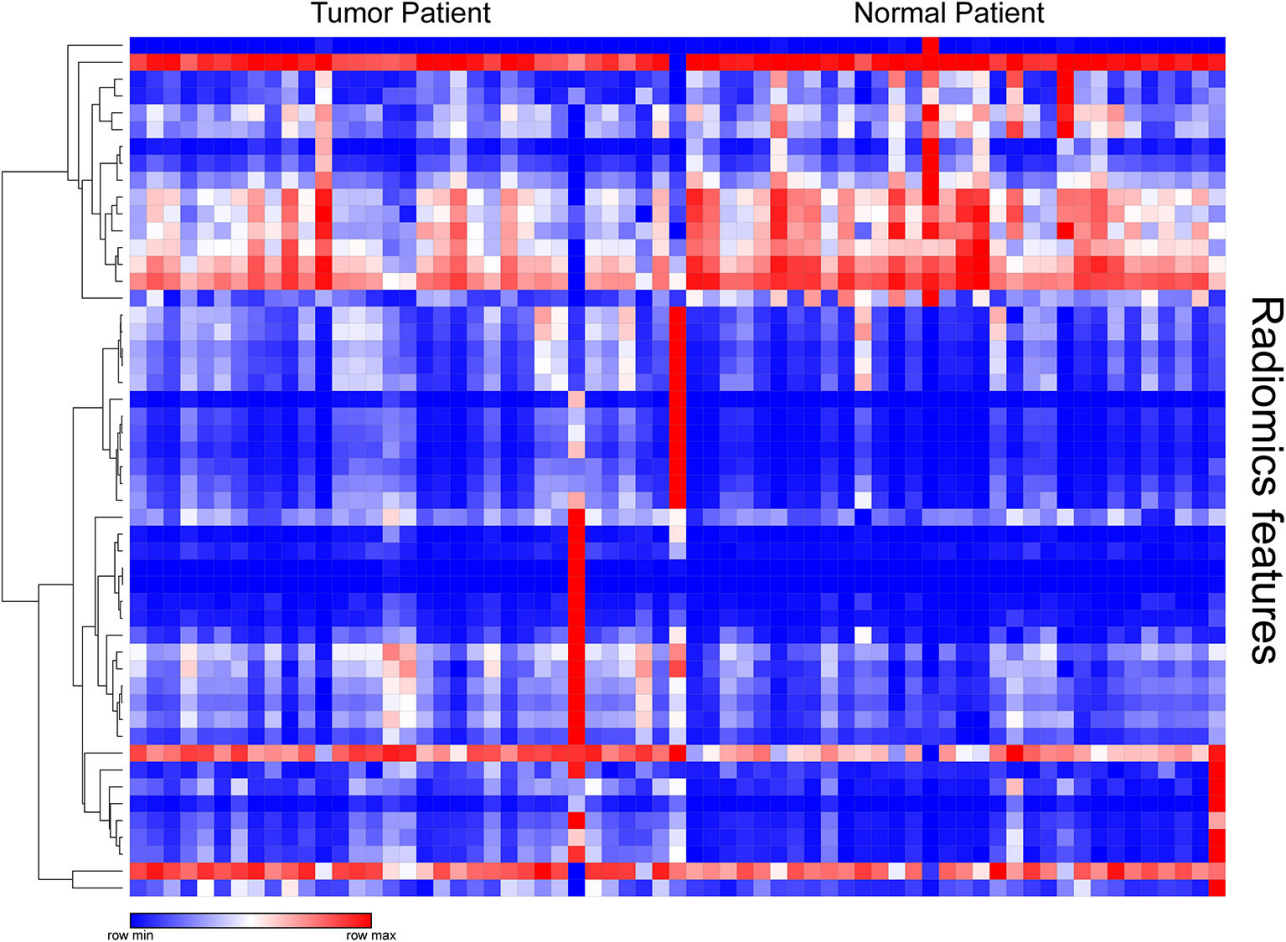
Heat map of texture parameters with differential significance between benign and malignant lesions in the training group.

After examining the normal distribution of the 72 texture features, we analyzed the differences between the two groups of texture parameters by Mann-Whitney *U*-test. Fifty-one texture parameters with different significance were selected and pre-processed. According to the heat map results, different colored partitions between the benign and malignant could be found (Figure 1).

For the texture parameters with statistically significant differences, we performed the ROC curve analysis. Finally, we took out the five parameters with diagnostic values better than SUVmax and performed correlation analysis on six parameters including SUVmax. Spearman's rank correlation coefficient was used to evaluate the correlation, and the results showed that SUV Variance was strongly correlated with SUV SD, so we eliminated SUV SD. The overall situation and distribution of the remaining five parameters in the training and testing groups are shown in Table 2. Finally, the optimal diagnostic threshold and its corresponding diagnostic sensitivity and specificity were found based on the Youden index (Table 3). The highest AUC value was the SUL peak, and the higher the value, the greater the degree of malignancy of the lesion, which is called a positive correlation, whereas the intensity value was inversely correlated with the degree of malignancy of the lesion.

**Table 2:** Differences in the overall distribution of parameters with good diagnostic value between the benign and malignant groups.

	**Training cohort**			**Test cohort**				
	**Tumor (*n* = 33) Median (IQR)**	**Normal (*n* = 32) Median (IQR)**	***P***	**Tumor (*n* = 12) Median (IQR)**	**Normal (*n* = 12) Median (IQR)**	***P***	***Pa***	***Pb***
SUL peak	5.97 (3.98, 8.10)	3.19 (2.70, 4.07)	0.001	6.82 (5.03, 8.61)	2.89 (2.49, 3.16)	0.001	0.603	0.095
Correlation	0.81 (0.74, 0.84)	0.69 (0.63, 0.74)	0.001	0.82 (0.78, 0.83)	0.62 (0.56, 069)	0.001	0.676	0.040
Intensity*	255.69 (247.10, 263.64)	278.10 (267.26, 289.10)	0.001	246.35 (234.49, 264.29)	258.00 (272.93, 297.01)	0.001	0.291	0.118
SUV Variance	2.42 (0.75, 4.61)	0.31 (0.21, 0.80)	0.001	3.23 (1.15, 5.89)	0.29 (0.22, 0.38)	0.001	0.568	0.328
Maximum SUV	10.06 (6.37, 12.69)	5.61 (4.50, 6.82)	0.001	11.64 (7.18, 14.31)	4.97 (4.45, 5.71)	0.001	0.409	0.131

*The Pa was derived from the Mann-Whitney U-test of tumor groups between the training and test cohorts and the Pb was derived from that of normal groups between the training and test cohorts*.

**Table 3:** AUC values of five texture parameters and associated diagnostic performance parameters incorporated into the model.

**Texture feature**	**AUC**	**CI 95%**	**Cut-off**	**Se (%)**	**Sp (%)**
SUL peak	0.831	0.728, 0.935	3.582	90.9	65.6
Correlation	0.827	0.719, 0.935	0.738	84.8	75.0
Intensity*	0.820	0.715, 0.925	263.577	75.8	81.2
SUV variance	0.817	0.709, 0.926	0.649	78.8	75.0
Maximum SUV	0.806	0.696, 0.915	7.40	69.7	84.4

### Diagnostic Modeling and Performance Analysis

**Figure 2:**
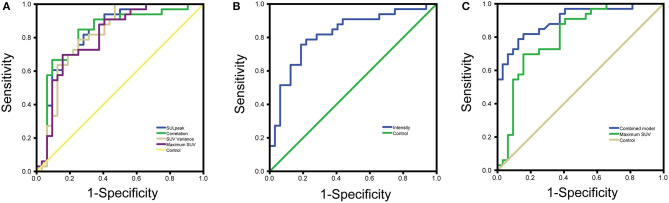
**(A)** ROC curves for four parameters with good diagnostic value **(B)** ROC curves for texture analysis parameters with inverse proportion of size and degree of malignancy **(C)** ROC curves for combined model diagnostic parameters vs. maximum SUV.

The jointly selected texture parameters were used to build a diagnostic model from the training group, which was constructed using three machine learning methods, logistic regression, decision tree, and support vector machine. The accuracy of classification in the test set was 87.5, 83.34, and 75%, respectively. The accuracy of the manual diagnosis was 84.27%. The combined model from logistic regression was used to plot the ROC curve with the probability output from the training group, and an AUC = 0.902 (95% CI = 0.803–0.962) was calculated. The DeLong test was used to compare the ROC curves of the combined model and SUVmax by logistic regression, and the results showed that the ROCs of the two groups were different (*P* = 0.0345), indicating that the diagnostic model constructed jointly with the texture analysis parameters had a better diagnostic value than SUVmax (Figure 2) (9).

### Survival Analysis

**Figure 3:**
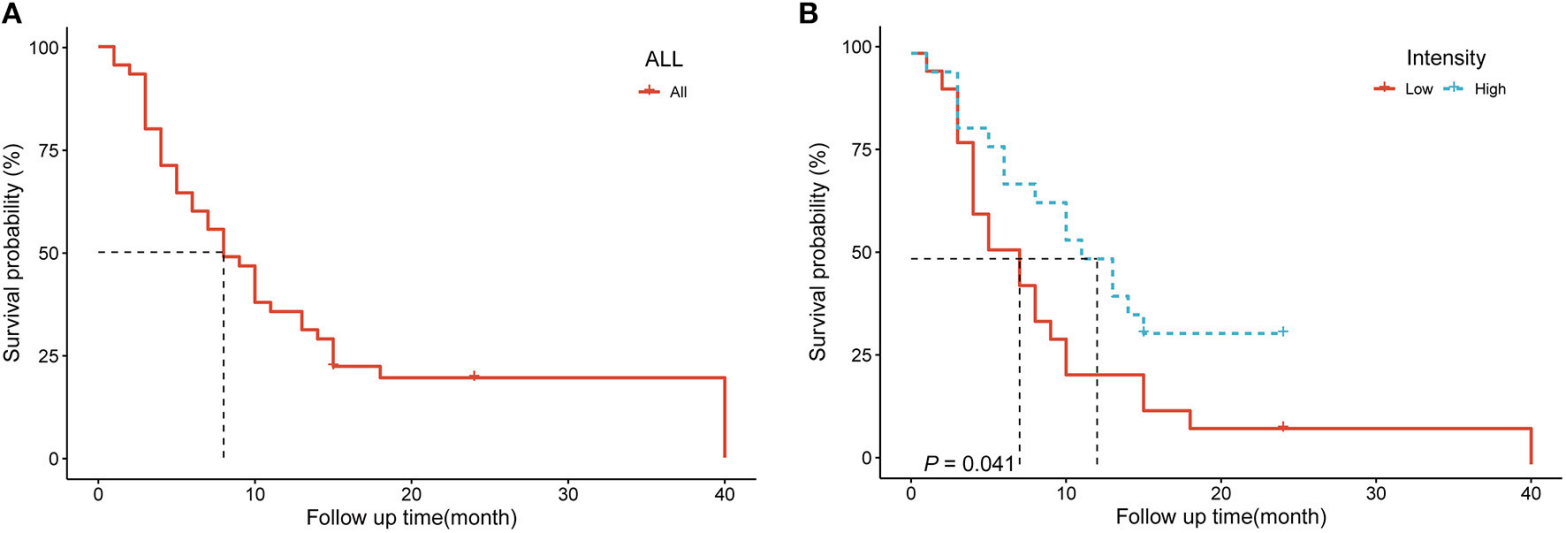
**(A)** Survival analysis curves for 45 patients with spinal metastases **(B)** Survival analysis of patients in different intensity groups.

The 45 patients with spinal metastases were followed for 3–40 months, with a median follow-up time of 8 months. By the last follow-up, 37 of the 45 patients with spinal metastases had died, and the median survival time for patients with metastatic spinal tumors was 8 months. The 1- and 2-year survival rates of patients were 48.9 ± 7.5% and 19.4 ± 6.0%, respectively (Figure 3). We performed a single-factor survival analysis of the five texture parameters and survival by classifying the five texture parameters into low and high groups based on the median of the texture parameters as the criterion, and the results showed that patients in the low intensity group had shorter survival (*P* = 0.041) (Figure 3). The multi-factor survival analysis showed that none of the five texture parameters and survival outcomes were statistically significant (*P* > 0.05).

## Discussion

Our study demonstrates the significance of PET/CT texture analysis for the analysis of spinal positive FDG uptake lesions for the identification and diagnosis of spinal metastases. We selected five texture parameters of diagnostic significance, four with a better diagnostic performance than current diagnostic modalities, and established a diagnostic model with a greater diagnostic performance than manual diagnosis. It shows the texture analysis can assist in the differential diagnosis spinal metastases and may have better diagnostic value than current methods.

There are two highlights of our study. Firstly, all the patients enrolled were patients with spinal metastases confirmed by definite pathological findings or follow-up results, and complete follow-up data on survival time were available. Secondly, to the best of our knowledge, this is the first time report that PET texture analysis has been used in the diagnosis of spinal metastases.

Radiomics is achieved progressively through segmentation of lesions, feature data extraction, database creation, and analysis of individualized data, while texture analysis is a class of feature data extraction with objective descriptive features. Most of the traditional imaging distributions of lesions are subjectively described, and correlating the results of texture analysis with the subjectively described features can make the diagnostic conclusions more convincing. ^18^F-FDG PET/CT texture analysis can provide more detail regarding tumor spatial information and tumor heterogeneity than clinically used parameters such as SUVmax, metabolic tumor volume (MTV), and total lesion glycolysis (TLG). Although PET/CT is superior to bone scan and CT for the diagnosis of spinal metastases (10), it is currently still prone to false positives for the diagnosis of spinal metastases. The SUVmax of Schmorl's nodes is similar to that of spinal metastases (11). High FDG uptake also occurs at different times after a benign fracture, so false-positive results may occur when performing ^18^F-FDG PET/CT imaging to assess metastases, although different uptake modalities and clinical correlations usually allow accurate differentiation of fractures from skeletal metastases (12). In addition, spinal metastases need to be differentiated from discontinuous spinal tuberculosis and spinal degenerative diseases (13, 14).

The robustness of the texture analysis software we use to measure texture values has been verified previously (8). PET/CT texture analysis has been shown to be effective in diagnosing and predicting prognosis in a variety of diseases. Bianconi et al. found a significant correlation between PET features, CT features, and histological type in non-small cell lung cancer (NSCLC) and texture analysis shows the potential for differentiating histological types in NSCLC (15). Feliciani et al. found that texture analysis of ^18^F-FDG PET has predictive value for the effectiveness of treatment of primary head and neck squamous cell carcinoma (HNSCC) treated with concurrent chemoradiotherapy (16). Lovinfosse et al. analyzed the SUVmax and mean standard uptake value (SUVmean), MTV, TLG, and 13 global, local, and regional texture features of 63 NSCLC patients undergoing stereotactic body radiotherapy (SBRT) who underwent ^18^F-FDG PET/CT prior to treatment. They found that differences in texture features measured at baseline ^18^F-FDG PET/CT appeared to be a strong independent predictor of prognosis in SBRT-treated NSCLC patients (17). Xu et al. found that the texture fractionation method of PET is useful for the differential diagnosis of benign and malignant bone and soft tissue lesions, in which the texture parameters coarseness and entropy have better diagnostic performance than SUV (18).

Gao et al. extracted the texture parameters from PET/CT images to build a support vector machine that can identify benign and malignant mediastinal lymph nodes in NSCLC patients (19). Oh et al. successfully evaluated the efficacy and survival of 70 patients with hypopharyngeal cancer after radiotherapy and chemotherapy by roughness in pre-treatment texture parameters (20). Pyka et al. found that texture analysis on PET images not only allowed some assessment of the local recurrence of NSCLC patients after radiotherapy, but also predicted their long-term survival (21).

Most tumors develop spinal metastases earlier; therefore, the characteristics of spinal metastases correlate with the overall survival prognosis of patients. The survival time of patients with metastases is related to a greater degree with the primary tumor and the location and number of metastases. Moreover, the only set of texture parameters with low intensities was found in the single-factor analysis. Patients in low intensity group had a worse prognosis, and multi-factor Cox regression analysis remained insignificant. It is possible that this texture parameter can be used to predict patient prognosis, but a large, prospective study is needed for further validation. The pathological diagnosis of spinal metastases in clinical practice is limited by inadequate access to tissue; it is more traumatic and unacceptable to most patients. Currently, there are three main approaches to the treatment of spinal metastases, chemotherapy, radiation therapy, and surgery. The goals of both medical and surgical treatment of metastases are to maximize the improvement in quality of life. Once a diagnosis of metastasis is established, the role of surgery or surgery in combination with other treatments can relieve pain, improve or maintain neurological function, and restore the structural integrity of the spine (22, 23).

### Limitations

There are certain limitations to our study. Only the relationship between texture analysis and character of the disease was analyzed, which can be followed by further refinement of the molecular type of the disease and the classification of the serologic examination indexes to evaluate the value of texture analysis. In addition, this is a single-center study, and the data from different centers may have some influence on the stability of texture analysis due to the different methods of PET image reconstruction, which can be used to study large samples of texture analysis data according to different machines and diseases to explore the stability value of texture analysis.

## Conclusions

We evaluated the feasibility of using texture analysis for the differential diagnosis of spinal metastases by analyzing and quantifying the overall distribution and heterogeneity of high FDG uptake spinal lesions, which can be effective in the diagnosis of spinal metastases. By carefully and objectively selecting out the PET texture parameters and deriving the optimal threshold to diagnose spinal metastases, certain texture features showed better diagnostic value than SUVmax. Thus, texture analysis in ^18^F-FDG PET/CT images may play a role in the differential diagnosis of spinal metastases, which provide more accurate and comprehensive guidance to clinical treatment.

## Data Availability Statement

The raw data supporting the conclusions of this article will be made available by the authors, without undue reservation.

## Ethics Statement

The studies involving human participants were reviewed and approved by Institutional Review Board of Shanghai Tenth People's Hospital. Written informed consent for participation was not required for this study in accordance with the national legislation and the institutional requirements.

## Author Contributions

XF, HZ, YY, SQ, XZ, and FY: conception and design. XF: acquisition, statistical analysis, or interpretation of the data. All authors: drafting of the manuscript, reviewed, and approved the final version of the manuscript.

## Conflict of Interest

The authors declare that the research was conducted in the absence of any commercial or financial relationships that could be construed as a potential conflict of interest.
